# Validating Insomnia Severity Index (ISI) in a Bangladeshi Population: Using Classical Test Theory and Rasch Analysis

**DOI:** 10.3390/ijerph19010225

**Published:** 2021-12-25

**Authors:** Mohammed A. Mamun, Zainab Alimoradi, David Gozal, Md Dilshad Manzar, Anders Broström, Chung-Ying Lin, Ru-Yi Huang, Amir H. Pakpour

**Affiliations:** 1 CHINTA Research Bangladesh, Savar, Dhaka 1342, Bangladesh; mamunphi46@gmail.com; 2 Department of Public Health and Informatics, Jahangirnagar University, Savar, Dhaka 1342, Bangladesh; 3 Social Determinants of Health Research Center, Research Institute for Prevention of Non-Communicable Diseases, Qazvin University of Medical Sciences, Shahid Bahonar Blvd, Qazvin 3419759811, Iran; zainabalimoradi@yahoo.com; 4 Department of Child Health and the Child Health Research Institute, The University of Missouri School of Medicine, Columbia, MO 65201, USA; gozald@health.missouri.edu; 5 Department of Nursing, College of Applied Medical Sciences, Majmaah University, Majmaah 11952, Saudi Arabia; m.manzar@mu.edu.sa; 6 School of Health and Welfare, Jönköping University, P.O. Box 1026, SE-55111 Jonkoping, Sweden; anders.brostrom@ju.se (A.B.); amir.pakpour@ju.se (A.H.P.); 7 Department of Clinical Neurophysiology, Linköping University Hospital, SE-58183 Linkoping, Sweden; 8 Institute of Allied Health Sciences, National Cheng Kung University Hospital, College of Medicine, National Cheng Kung University, Tainan 701401, Taiwan; 9 Biostatistics Consulting Center, National Cheng Kung University Hospital, College of Medicine, National Cheng Kung University, Tainan 701401, Taiwan; 10 Department of Occupational Therapy, College of Medicine, National Cheng Kung University, Tainan 701401, Taiwan; 11 Department of Family and Community Medicine, E-DA Hospital, Kaohsiung 82445, Taiwan; 12 School of Medicine for International Students, College of Medicine, I-Shou University, Kaohsiung 82445, Taiwan

**Keywords:** Bangladesh, COVID-19, insomnia, psychometric testing, psychological distress

## Abstract

The COVID-19 outbreak is associated with sleep problems and mental health issues among individuals. Therefore, there is a need to assess sleep efficiency during this tough period. Unfortunately, the commonly used instrument on insomnia severity—the Insomnia Severity Index (ISI)—has never been translated and validated among Bangladeshis. Additionally, the ISI has never been validated during a major protracted disaster (such as the COVID-19 outbreak) when individuals encounter mental health problems. The present study aimed to translate the ISI into Bangla language (ISI-Bangla) and validate its psychometric properties. First, the linguistic validity of the ISI-Bangla was established. Then, 9790 Bangladeshis (mean age = 26.7 years; SD = 8.5; 5489 [56.1%] males) completed the Bangla versions of the following questionnaires: ISI, Fear of COVID-19 Scale (FCV-19S), and Patient Health Questionnaire-9 (PHQ-9). All the participants also answered an item on suicidal ideation. Classical test theory and Rasch analyses were conducted to evaluate the psychometric properties of the ISI-Bangla. Both classical test theory and Rasch analyses support a one-factor structure for the ISI-Bangla. Moreover, no substantial differential item functioning was observed across different subgroups (gender, depression status (determined using PHQ-9), and suicidal ideation). Additionally, concurrent validity of the ISI-Bangla was supported by significant and moderate correlations with FCV-19S and PHQ-9; known-group validity was established by the significant difference of the ISI-Bangla scores between participants who experienced suicidal ideation and those without. The present psychometric validation conducted during the COVID-19 outbreak suggests that the ISI-Bangla is a promising and operationally adequate instrument to assess insomnia in Bangladeshis.

## 1. Introduction

Insomnia as well as other sleep problems, are intrinsically linked to people’s wellness, health, and development [1,2,3,4,5,6]. Studies have shown fear of COVID-19, a pandemic caused by the novel coronavirus (SARS-CoV-2) as a potential threat to good sleep and possibly linked to psychological distress and suicidality [7,8,9]. Indeed, COVID-19 has been associated with dramatic increases in the prevalence of psychosocial health problems in light of its ability to rapidly spread, induce severe multi-organ symptoms, and with elevated mortality rates. Furthermore, even among survivors, COVID-19 can impair physical health, especially among those suffering from chronic diseases and among the older people [10]. Although several vaccines are available for COVID-19, the seriousness of COVID-19 pandemic is far away from being under control [11]. Thus, mental health problems are frequent and are either induced by COVID-19 among those infected or among the general population by virtue of the lockdown measures and associated economic hardship imposed by the various social and work-related restrictions [10]. Among mental health problems, insomnia has emerged as a latent—yet frequent—issue worldwide [12].

Therefore, improved understanding and detection of insomnia and its severity is an important issue for healthcare providers, and such challenges are particularly relevant during a tough time such as the one imposed by the COVID-19 pandemic. Indeed, the problem of insomnia is a potentially serious issue for Bangladeshi populations, given the high levels of COVID-19-related fear and suicidality [13]. Furthermore, since insomnia is also highly associated with psychological distress, such as fear and suicidal ideations [14,15], Bangladeshi healthcare providers need to have a simple, valid, and readily implementable tool to identify individuals at risk for insomnia.

The Insomnia Severity Index (ISI) is a commonly used and extensively validated instrument to screen for insomnia and to assess its severity level worldwide [16,17,18]. To the best of our knowledge, the ISI has not been translated into the Bangla language and validated for the Bangladeshi population, a pressing requirement considering the current COVID-19 pandemic impact on the country.

Two theories of psychometric testing have been proposed to be used together for validation of any particular survey instrument and will enable healthcare providers and users to have a better understanding of the psychometric features of any instrument they use in their practice [19]. More specifically, psychometric testing using the Classical Test Theory (CTT) has the benefits of easy understanding and being familiar to healthcare providers [19]. Thus, the CTT has been widely used in the literature [20,21] and further encouraged by the U.S. Food and Drug Administration (FDA) [22]. In addition to CTT, the Rasch analysis from the modern test theory has other advantages during validation of a survey instrument, particularly when evaluating individual item validity (i.e., whether an individual item is well embedded in its belonging concept) and estimating item difficulty using interval scale (i.e., the difficulty of each item has the addition feature) [23]. In the context of instrument development and validation, we should stress that translated language versions of existing validated tools need to undergo their own validation process and be aligned with the conceptual-practice model (e.g., using the insomnia concept to design the ISI for assessing insomnia severity) [24]. Considering that the ISI has never been translated into Bangla and the potential impacts of the COVID-19 pandemic on Bangladeshis’ sleep quality, the present study aimed to translate the ISI into Bangla (named as the ISI-Bangla) and validate this version of ISI in a large Bangladeshi cohort during COVID-19 pandemic.

## 2. Materials and Methods

### 2.1. Participants, Procedure, and Ethics

The present cross-sectional study was carried out between 1 and 10 April 2020. The target population was the Bangladeshi population. The online survey was conducted via social media and online blogs, and a total of 10,067 participants completed the survey. After removing non-adults (<18 years of age), 9790 completed surveys were retained for final analysis (mean age: 26.7 ± 8.52 years [SD]). The study received ethical approval from the Institute of Allergy and Clinical Immunology of Bangladesh, Dhaka, Bangladesh (IRBIACIB/CEC/03202005) and from Jahangirnagar University, Dhaka, Bangladesh (BBEC, JU/M 2O20/COVlD-l9/ (9)2) ethics committees. An online consent form was required for all adult participants. All the participants could only complete the survey once because our web-based monitoring system can detect and track if a participant completes twice. Moreover, the online survey clearly instructed participants to fill up the questionnaire only once.

### 2.2. Translation Process of the ISI-Bangla

A forward-backward translation method was used to adjudicate the ISI into Bangla in a culturally-adapted fashion [25], similar to the approach used to translate Fear of COVID-19 Scale into Bangla version [26]. Three independent translators—two subject matter experts (i.e., public health) and the other being highly experienced in cultural and linguistic distinctions in English and Bangla—translated the seven-item scale into Bangla (i.e., forward translation). Both of the versions were compiled and further translated back into English by another professional translator with medical translation proficiency and by a bilingual individual who had not previously seen the English version of the ISI (i.e., back translation). After compiling the back translated versions, all versions were compared and submitted to an expert panel of five members (they were all experts in public health with two had extensive research experience in sleep and one had extensive research experience in psychometrics). The panel scrutinized and finalized the items with the seven questions being retained. Pre-testing was then conducted among 140 individuals using online platforms across different age groups. The research team then implemented the suggested changes derived from such initial testing. The final version of the questionnaire was then administered during the large-scale study.

### 2.3. Measures

#### 2.3.1. Insomnia Severity Index-Bangla (ISI-Bangla)

The ISI includes seven items to assess an individual’s insomnia severity. With the use of a five-point Likert scale (0 = none, very satisfied, not at all noticeable, not at all worried, or not at all interfering; 4 = very severe, very dissatisfied, very much noticeable, very much worried, or very much interfering), the ISI total score ranges from 0 to 28, and a higher score indicates a more severe level of insomnia. More specifically, an ISI total score higher than 7 indicates the presence of depression [27]. As part of this study, the ISI was translated into the Bangla version, i.e., the ISI-Bangla.

#### 2.3.2. Fear of COVID-19 Scale (FCV-19S)

The Bangla version of the FCV-19S includes seven items to assess an individual’s fear of COVID-19. With the use of a five-point Likert scale (1 = strongly disagree; 5 = strongly agree), the FCV-19S total score ranges from 7 to 35 and a higher score indicates a higher level of fear [28]. The FCV-19S has been translated into the Bangla version with satisfactory psychometric properties [26].

#### 2.3.3. Patient Health Questionnaire-9 (PHQ-9)

The Bangla version of the PHQ-9 includes nine items to assess an individual’s depression level. With the use of a four-point Likert scale (0 = not at all; 3 = nearly every day), the PHQ-9 total score ranges from 0 to 27, and a higher score indicates a higher level of depression. More specifically, a PHQ-9 total score higher than 9 indicates the presence of depression [29]. The PHQ-9 has been translated into the Bangla version with satisfactory psychometric properties.

#### 2.3.4. Suicidal Ideation

One item was used to assess the suicidal ideation of the participants. The item question is, “Do you think about committing suicide and are these thoughts persistent and related to COVID-19 issues?” with a dichotomous response scale (yes vs. no) similar to previous research [29,30].

### 2.4. Data Analysis

Descriptive statistics were used to summarize participants’ characteristics, including age, gender, health behaviors, self-perceived health status, suicidal ideation, and scores in ISI, FCV-19S, and PHQ-9.

Psychometric testing of CTT was then applied to understand the item properties (factor loadings derived from a one-factor model of confirmatory factor analysis (CFA) and item-total correlations) and scale properties (ceiling and floor effects, internal consistency, CFA, average variance extracted, and composite reliability) of the ISI-Bangla. The acceptable values for these properties were: factor loadings and item-total correlations above 0.4; ceiling and floor effects below 20%; internal consistency via Cronbach’s α over 0.7; CFA fit indices of comparative fit index (CFI) and Tucker–Lewis index (TLI) exceed 0.9; CFA fit indices of root-mean square error of approximation (RMSEA) and standardized root mean square residual (SRMR) less than 0.08; average variance extracted more than 0.5; and composite reliability larger than 0.6 [31,32]. CFA was conducted using the diagonally weight least squares (DWLS) estimator.

Psychometric testing via Rasch model was also applied to understand the item properties (infit and outfit mean squares (MnSq) and differential item functioning (DIF) across gender, depression status, and suicidal ideation) and scale properties (item separation reliability, item separation index, person separation reliability, and person separation index) of the ISI-Bangla. The acceptable values for these properties were: infit and outfit MnSq between 0.5 and 1.5; DIF contrast lower than 0.5; item separation reliability and person separation reliability over 0.7; and item separation index and person separation index larger than 2 [33,34,35]. Table 1 further summarizes the interpretations of the cutoffs used for the psychometric testing.

All statistical analyses were conducted using IBM SPSS 24.0 (IBM Corp., Armonk, NY, USA) for descriptive statistics and classical test theory analyses. In addition, the lavaan package in R software (Vienna, Austria) [36] was used for CFA analyses, while WINSTEPS were used for Rasch analysis [37]. Two-way *p*-value < 0.05 was considered to achieve statistical significance.

## 3. Results

Recruited participants (*n* = 9790) had a mean age of 26.7 ± 8.5 years, with slightly more than half being males (*n* = 5489; 56.1%). Most of the participants were single (*n* = 6913; 70.6%), non-smokers (*n* = 8331; 85.1%), did not consume alcoholic beverages (*n* = 9528; 97.3%), and reported fair to good self-perceived health (*n* = 9484; 96.9%). However, 5% of the participants (*n* = 494) had a suicidal ideation. A descriptive summary of participants’ characteristics and the ISI-Bangla, FCV-19S, and PHQ-9 scores are presented in Table 2 and Table 3. Moreover, both Bangla versions of FCV-19S and PHQ-9 had satisfactory internal consistency (Cronbach α = 0.875 for FCV-19S and 0.820 for PHQ-9).

The item properties of the ISI-Bangla are shown in Table 4. The results computed using CTT showed that the factor loadings derived from the CFA were strong (0.626 to 0.780), and the item-total correlations were satisfactory (0.584 to 0.734). Among the computed findings using the Rasch model, MnSq values were adequate (infit MnSq = 0.67 to 1.23; outfit MnSq = 0.73 to 1.20), DIF contrasts were not substantial (DIF contrasts across gender = −0.25 to 0.30; DIF contrasts across depression status = −0.28 to 0.27; DIF contrasts across suicidal ideation = −0.27 to 0.17).

The scale properties of the ISI-Bangla are presented in Table 5. Using the CTT, ceiling (9.4%) and floor effects (0.2%) were low; internal consistency (α = 0.876) together with average extracted variance (0.51) and composite reliability (0.88) were adequate; and the fit indices of the CFA (CFI = 0.990; TLI = 0.985; RMSEA = 0.055; SRMR = 0.046) were satisfactory. Using the Rasch analysis, the item separation properties (reliability = 1.00; index = 31.64) were promising, and the person separation properties (reliability = 0.74; index = 1.70) were nearly acceptable.

Moreover, the concurrent validity of the ISI-Bangla was also evaluated and showed significant and moderate correlations with FCV-19S (*r* = 0.36; *p* < 0.001) and PHQ-9 (*r* = 0.58; *p* < 0.001). Known group validity of the ISI-Bangla was supported by the significant difference of ISI-Bangla scores between participants who had a suicidal ideation (mean [SD] ISI score = 12.81 [6.93]) and those who had no suicidal ideation (mean [SD] ISI score = 7.69 [6.10]; *t* = 18.06; *p* < 0.001; Cohen’s d = 0.78).

## 4. Discussion

An effective and feasible instrument to assess insomnia can help healthcare providers identify and ultimately manage such a frequent and health-detracting medical problem. Insomnia is also more likely to emerge during protracted adverse events, and the recent COVID-19 pandemic clearly has illustrated such a trend [12,38]. In light of the adverse consequences associated with the presence of insomnia, and the opportunities afforded by simple and valid instruments, such as the ISI to diagnose and manage this condition, it seemed of great importance and urgency to enable this tool for Bangladesh. Therefore, the present study translated and subsequently examined the psychometric properties of ISI (ISI-Bangla version), the commonly used instrument on insomnia [16,17]. The ISI has the advantage of using standard and diagnostic criteria to define insomnia severity, which is outlined in the International Classification of Sleep Disorders (ICSD) and the fourth edition of the Diagnostic and Statistical Manual of Mental Disorders (DSM-5) [39,40]. Since the ISI has never been validated in a Bangla version, we took advantage of a period with a major and protracted disaster with the COVID-19 pandemic to perform this appraisal. With the use of two testing theories (CTT and Rasch), the present study found that the ISI-Bangla exhibits promising properties in both item and scale levels. Therefore, the results concurred robustly with the prior findings reported by others on the ISI psychometric properties [16,18,40,41], and further extend on such prior findings by confirming that the ISI-Bangla remains effective during a disaster period such as the one imposed by the COVID-19 pandemic. The promising properties of the ISI-Bangla as identified in the present study are comparable to other language versions of the ISI, such as the Chinese and Persian versions [16,42]. For example, the Cronbach α in the present study is 0.876, which is comparable to the reported Cronbach α of 0.830 in the study by Chung and colleagues and the reported ω of 0.79 in the study by Lin and collaborators. However, the literature reports different factor structures for the ISI ranging from one to three factors [42,43,44], while both CFA and Rasch results in the present study support the use of a one-factor structure. The factor structure of an instrument may vary because of differences in the characteristics of the cohort sample [35], and disparities/discrepancies in the methodology of factor analysis that were used in different studies. Therefore, more evidence on the factor structure of the ISI-Bangla is warranted to corroborate the findings of the one-factor structure in the present study.

The potential benefit of ascertaining the one-factor structure for the ISI-Bangla through future research is that healthcare providers can then use a simple concept (i.e., insomnia) to assess its presence and severity among all individuals, and then proceed with confidence to diagnose and treat those suspected to have insomnia problems [16]. In the context of the pandemic, healthcare providers may then use the tool to gain some notion as to who are the vulnerable individuals who are at risk of developing insomnia or who actually has insomnia. As such, using the ISI-Bangla may also guide healthcare providers in Bangladesh to screen and detect insomnia issues, and take early action to deal with this sleep problem or with insomnia concurrent with other co-morbid disorders [45]. Indeed, using the ISI in a large population of US Veterans revealed substantial outcomes and financial benefits [45]. Therefore, we can expect similar benefits can be gained if the ISI-Bangla undergoes widespread implementation within the healthcare system.

There are several strengths in the present study that are worth mentioning. First, the representativeness of the present sample is high in light of the robust recruitment method that was used: residents from all the 64 Bangla districts have been approached with each district having participants recruited in the present study. Second, the translation procedure of the ISI-Bangla in the present study follows common standard procedural guidelines [25]. Therefore, the linguistic validity of the ISI-Bangla is ensured. Third, advanced psychometric testing, including CFA and Rasch models, were applied to examine different aspects of psychometric properties of the ISI-Bangla. The psychometric evidence for the ISI-Bangla is thus comprehensive and thorough. However, there are also some limitations in the present study. First, a cross-sectional design was used in the present study and thus the causal relationships between the tested concepts (i.e., fear of COVID-19, depression, suicidal ideation, and insomnia) cannot be inferred with certainty. This type of design can only provide the associated information between these studied variables, but not their sequences (i.e., which variables happened early and which later). Second, an online survey was used in the present study to collect data. Therefore, common limitations of using this data collection method include sample representativeness (i.e., those without internet access were unable to participate) and self-reported biases (e.g., the recall bias, common method variance bias, and social desirability bias) that cannot be controlled. Lastly, the collected sample in the present study was relatively young, and the present results’ generalizability is thus limited.

## 5. Conclusions

In summary, the present study conducted during the COVID-19 outbreak period enabled translation and validation of the ISI-Bangla, and such a process indicates that this tool emerges as a promising and feasible instrument to assess insomnia for Bangladeshis. Furthermore, the ISI-Bangla showed the ability to effectively assess insomnia severity for Bangladeshis in a difficult period such as the one imposed by the pandemic. Such assumptions should, however, undergo additional corroboration in selected clinical cohorts. Notwithstanding, healthcare providers should be able to use the ISI-Bangla to screen for insomnia among all those adult Bangladeshis seeking medical care, thereby enabling earlier diagnosis and treatment for this highly prevalent condition.

## Figures and Tables

**Table 1:** Interpretations of the cutoffs used in the psychometric testing.

Fit Indices	Possible Range	Suggested Cutoff
**Classical Test Theory**		
Ceiling effects (%)	0–100	<20
Floor effects (%)	0–100	<20
Cronbach’s α	0–1	>0.7
Comparative fit index in CFA	0–1	>0.9
Tucker–Lewis index in CFA	0–1 ^a^	>0.9
Root-mean square error of approximation in CFA	0–1	<0.08
Standardized root mean square residual in CFA	0–1	<0.08
Average variance extracted in CFA	0–1	>0.5
Composite reliability in CFA	0–1	>0.6
**Rasch Analysis**		
Item separation reliability	0–1	>0.7
Item separation index	>0	>2
Person separation reliability	0–1	>0.7
Person separation index	>0	>2
Infit and outfit mean square (MnSq)	−∞ to ∞	0.5 to 1.5
Differential item functioning	−∞ to ∞	−0.5 to 0.5

^a^ In some occasions, the Tucker–Lewis index is beyond 1; CFA = confirmatory factor analysis.

**Table 2:** Participant characteristics of categorical data in the present sample (*n* = 9790).

Variable	*n* (%)
Gender	
Gender; Male	5489 (56.1)
Gender; Female	4286 (43.8)
Gender; Others	15 (0.2)
Marital status	
Marital status; Single	6913 (70.6)
Marital status; Married	2759 (28.2)
Marital status Others	118 (1.2)
Current smoker; No	8331 (85.1)
Current smoker; Yes	1459 (14.9)
Current alcoholic beverage consumer; No	9528 (97.3)
Current alcoholic beverage consumer; Yes	262 (2.7)
Suicidal ideation; No	9296 (95.0)
Suicidal ideation; Yes	494 (5.0)
Current health status; Poor	306 (3.1)
Current health status; Fair	2714 (27.7)
Current health status; Good	6770 (69.2)

**Table 3:** Participant characteristics of scale data in the present sample (*n* = 9790).

Scale and Items	Mean (SD)	Cutoff for Presence
**Fear of COVID-19 Scale Score (Range: 7–35)**	21.4 (6.0)	>16.5
1. Most afraid of Corona		
2. Uncomfortable to think about Corona		
3. Clammy hands when thinking about Corona		
4. Afraid of losing life because of Corona		
5. Become nervous or anxious when watching Corona news and stories		
6. Cannot sleep because of worrying about getting Corona		
7. Heart races or palpitates when thinking about getting Corona		
**Patient Health Questionnaire-9 (Range: 0–27)**	7.9 (5.1)	>9
1. Little interest or pleasure in doing things		
2. Feeling down, depressed, or hopeless		
3. Trouble falling/staying asleep, or sleeping too much		
4. Feeling tired or having little energy		
5. Poor appetite or overeating		
6. Feeling bad about yourself		
7. Trouble concentrating on things		
8. Moving or speaking so slowly		
9. Thoughts on dead or self-hurting		
**Insomnia Severity Index-Bangla (Range: 0–28)**	8.0 (6.2)	>7
1. Difficulty falling asleep		
2. Difficulty staying asleep		
3. Problems waking up too early		
4. Satisfaction with current sleep pattern		
5. Noticeable to others regarding sleep problems in terms of impairing the quality of life		
6. Worried/distressed about current sleep problem		
7. Sleep problem interfere with daily functioning currently		

**Table 4:** Psychometric properties of the Insomnia Severity Index (ISI) in item level.

Item #	Classical Test Theory Results		Rasch Analysis Results						
	Factor loading ^a^	Item-Total Correlation	Infit MnSq	Outfit MnSq	Difficulty	Discrimination	DIF Contrast Across Gender ^cd^	DIF Contrast Across Depression Status ^ce^	DIF Contrast Across Suicidal Ideation Status ^cf^
ISI-Bangla-1	0.712	0.660	1.10	1.04	−0.48	0.90	−0.06	−0.12	−0.04
ISI-Bangla-2	0.632	0.593	1.16	1.12	−0.25	1.17	−0.25	0.20	0.17
ISI-Bangla-3	0.645	0.600	1.23	1.09	0.43	0.92	0.00	0.14	0.13
ISI-Bangla-4	0.797	0.734	0.67	0.73	−0.67	0.90	−0.11	0.27	0.05
ISI-Bangla-5	0.626	0.584	1.23	1.20	0.56	0.90	0.30	−0.10	0.00
ISI-Bangla-6	0.780	0.722	0.86	0.77	0.39	1.12	0.07	−0.17	−0.04
ISI-Bangla-7	0.779	0.722	0.87	0.83	0.01	1.14	0.11	−0.28	−0.27

^a^ Based on confirmatory factor analysis; ^c^ DIF contrast > 0.5 indicates substantial DIF; ^d^ DIF contrast across gender = Difficulty for Females-Difficulty for Males; ^e^ DIF contrast across depression status = Difficulty for participants without any Depression-Difficulty for participants with depression; ^f^ DIF contrast across suicidal ideation status = Difficulty for participants without any suicidal ideation-Difficulty for participants with suicidal ideation; MnSq = mean square error; DIF = differential item functioning.

**Table 5:** Psychometric properties of the Insomnia Severity Index (ISI) in scale level.

Psychometric Testing	Value	Suggested Cutoff
Ceiling effects (%)	9.4	<20
Floor effects (%)	0.2	<20
Internal consistency (Cronbach’s α)	0.876	>0.7
Confirmatory factor analysis		
χ^2^ (*df*)	432.81 (14) *	--
Comparative fit index	0.990	>0.9
Tucker–Lewis index	0.985	>0.9
Root-mean square error of approximation	0.055	<0.08
Standardized root mean square residual	0.046	<0.08
Average variance extracted	0.51	>0.5
Composite reliability	0.88	>0.6
Item separation reliability from Rasch	1.00	>0.7
Item separation index from Rasch	31.64	>2
Person separation reliability from Rasch	0.74	>0.7
Person separation index from Rasch	1.70	>2

* *p* < 0.001.

## Data Availability

The data presented in this study are available on request from the corresponding author. The data are not publicly available due to the regulations made by the approval of the ethics committee.
